# Muscle Contraction and Force: the Importance of an Ancillary Network, Nutrient Supply and Waste Removal

**DOI:** 10.3390/ijms9081472

**Published:** 2008-08-20

**Authors:** Dagmar A. Brüggemann, Jens Risbo, Stefan G. Pierzynowski, Adrian P. Harrison

**Affiliations:** 1 Department of Food Science, Faculty of Life Sciences, Copenhagen University, Frederiksberg C, Denmark. E-Mails: dab@life.ku.dk (D. B.); jri@life.ku.dk (J. R.); 2 Department of Cell & Organism Biology, Lund University, Lund, Sweden. E-Mail: stefan.pierzynowski@cob.lu.se (S. P.); 3 Department of Animal & Veterinary Basic Science, Faculty of Life Sciences, Copenhagen University, Frederiksberg C, Denmark

**Keywords:** Collagen, muscle performance, fatigue, capillary network

## Abstract

Muscle contraction studies often focus solely on myofibres and the proteins known to be involved in the processes of sarcomere shortening and cross-bridge cycling, but skeletal muscle also comprises a very elaborate ancillary network of capillaries, which not only play a vital role in terms of nutrient delivery and waste product removal, but are also tethered to surrounding fibres by collagen ”wires”. This paper therefore addresses aspects of the ancillary network of skeletal muscle at both a microscopic and functional level in order to better understand its role holistically as a considerable contributor to force transfer within muscular tissue.

## 1. Introduction

### 1.1. Historical

The first account of the composition of body tissues is accredited to the presocratic Greek philosopher of Agrigentum, Empedocles (c. 490-430 BC). Empedocles maintained that all matter was made up of four elements, water, earth, air and fire, with various proportions of these giving rise to, for example, tendon, nerve, muscle and bone. Movements of limbs was believed to be the result of the psyche or soul, comprising the lightest and fastest moving particles of the body originating principally in the brain and communicating with the tissues acted upon.

It was not until the 2^nd^ and 3^rd^ centuries AD, however, that Galen of Pergamon (129 – c. 200 AD) and his students greatly refined the idea of movement. For instance, Galen knew that nerves arising in the brain and spinal cord were essential for sensation and motor action, for if they were cut or damaged, sensation and movement ceased [18]. Yet it was not until 1691, when Giorgio Baglivi (1668–1707) settled in Bologna as Malpighi’s assistant, that scientists began to see the body as a machine, and used microscopy to investigate nerves, smooth and striated muscles and even distinguish between different kinds of fibre [17]. Then in 1876 Latschenberg and Deahna, and then Gaskell in 1879 proposed that muscle vascular tone was inhibited by metabolites released during contractions [44].

Finally, in the mid 1950′s, with the publication of two papers in *Nature* came the concept of muscle contraction powered by the movement of one muscle filament sliding past another [21, 26]. In the past fifty years a great deal of progress has been made with many aspects of muscle contraction now being well studied and understood. However, the challenge of being able to apply the working knowledge of muscle physiology in a holistic fashion still remains largely unexplored.

### 1.2. Contemporaneous

If we consider the contractile machinery of muscle in the context of interrelated factors, then suddenly we are faced with a very complex area of study. At the one level it is essential that one can combine contractile units, that is to say muscle fibres, so as to augment and optimize force production, but at another level one needs also to provide space for an ancillary network to facilitate the supply of nutrients and the removal of waste compounds. It is this interconnection both between the anatomical and physiological parameters that comprise muscle and between the fundamental requirements of contraction *versus* the continued supply and removal of compounds that makes muscle contraction and force production so fascinating a subject to research and comprehend.

In its simplest form one could consider the interplay of muscle fibres, which may perhaps be seen as cables within the structure of muscle tissue, with that of the capillaries, which one might equally term as tubes, in association with the network of connective tissue that not only surrounds the fibres and capillaries but also acts as a cleavage plane between muscle layers, a structure that may perhaps be referred to as wires.

Connective tissue binds animal and human cells together. Moreover, the common link between all connective tissue is that their extracellular material contains collagen [29], which is important at a number of levels. The collagenous skeleton of the heart has been shown to play a very important role in the maintenance of the passive properties of myocardium [7, 25, 27]. The sheath wall of cardiac myocytes is formed by thin collagen fibres which continue into adjacent sheaths providing a continuous collagenous meshwork that is attached to and at the same time continuous with both the septum of the heart and the myocyte surface [27]. However, whilst this structure is fundamental to myocardial mechanics during contraction [50], ensuring an equal distribution of force during systole, it is worth bearing in mind that an excess of this particular structure, termed fibrosis, which is often found in the hearts of hypertensive individuals, results in an increase in collagen that impairs contractile capabilities and compromises heart function [27].

Clearly then, connective filaments, which we earlier referrred to as wires, play a fundamental role in muscle contraction. However, one should also consider a role for the connective tissue tethers in muscle, perhaps in terms of the deformation pattern of capillaries in contracting muscles. This is both a novel and an exciting area of research, although it has been known for some time that the capillary network supplying muscles has what appears to be a dual circulation with the secondary circulation favouring nutrient delivery to the septa and tendons [3]. The microcirculation of muscle plays a major role in the control of metabolism but a recent review and new evidence now support a control of blood flow within muscle that follow a nutritive and non-nutritive route [12]. In the mid 80′s Lindbom and Arfos revealed a vasculature in rabbit tenuissimus muscle that comprised transverse arterioles supplying both capillaries in muscle tissue proper and adjacent connective tissue [33]. Moreover, these two networks, the muscle capillaries and the adjacent connective tissue capillaries were found to be operating in parallel, with a flow distribution estimate that favoured only 10–20% of total muscle blood flow to surrounding connective tissue at rest [8].

It is precisely the interrelation between these so called cables, tubes and wires in muscle tissue, that is rarely addressed in a holistic perspective, particularly when aspects of contraction and force are being considered, that we wish to explore in this manuscript.

## 2. Results and Discussion

### 2.1. Collagen (wires)

The traditional way of looking at connective tissue in skeletal muscle focuses primarily on the structural integrity of muscle, which is thought to be maintained by three layers of intramuscular connective tissue: the endomysium that surrounds the individual muscle fibre, the perimysium that bundles a group of muscle fibres and the epimysium that enfolds the whole muscle [43]. Moreover, since the most plentiful and widespread protein component of the connective tissue is collagen, there has been a great deal of focus on the identification and localisation of collagenous structures [41]. The structure and function of collagen in mammalian species has now been widely studied and a total of 27 collagen types have been identified [6]. The fibril forming collagen types I, III and V are the main constituents in mammalian skeletal as well as fish swimming muscle [2, 46]. In addition, the basement membrane collagen IV and the microfibrillar collagen VI have been identified in both classes [10, 32]. Other non-fibrillar collagens present in mammalian skeletal muscle, are the FACIT collagen types XII and XIV [11, 34]. Interestingly, all of these collagen types are also part of the vascular wall or the peripheral nervous system [19, 30], which are all tissues that are highly interconnected in muscle.

**Figure 1.**
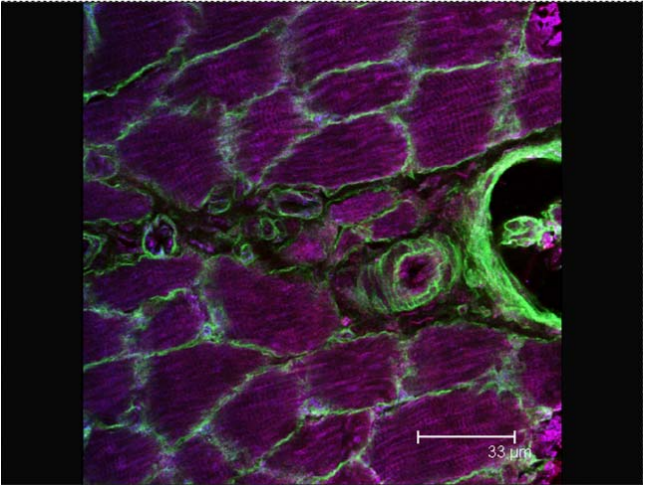
CLSM microscope image through a rat hind leg muscle, showing different hierarchies of vessels in the perimysium using anti–Laminin (green) and Phalloidin against F-Actin (blue) staining. Note the differences in composition of the walls of venules, arterioles and capillaries, as well as the differences in their diameter. Scale bar = 33 μm.

In Figure 1, a CLSM image shows the abundance of different hierarchies of vessels in the perimysium using anti Laminin-immune labeling in combination with Phalloidin staining for F-Actin. Laminin is a basement membrane protein and collagen receptor. Filamentous Actin is not only found in skeletal muscle cells but also in the cytoskeleton of smooth muscle and other cells. Using these molecular markers different vessel types can be distinguished by the very nature of their wall composition. The venule wall contains only a few smooth muscle cells and shows a much stronger laminin staining than the arterioles, which have a relatively thick tunica media and weaker laminin labeling.

In a study of vascular development in rat foreleg extensor muscle of 12-day-old embryos up to 9 days post-partum, light microscopy analysis of semi-thin plastic sections, showed a change in vasculature that was closely related to functional demands [5]. Early in differentiation of muscles the epimysium was found to be covered by a netlike capillary plexus, and later with the establishment of the perimysium on day 16 of embryonic development some capillaries were visible within the muscle fibre bundles, and than finally, by day 9 post-partum the vascular system was found to resemble that of adult muscle. Clearly, it can be concluded from the work of Bogusch [5], that the process of vascular development is one that progresses from the periphery of fibres to a greater degree of infiltration into smaller and smaller compartments within muscles as the fibres hyperthrophy and require a greater degree of vascularization deep within their core, something that occurs in close correlation to the establishment of the muscular connective tissue.

**Figure 2.**
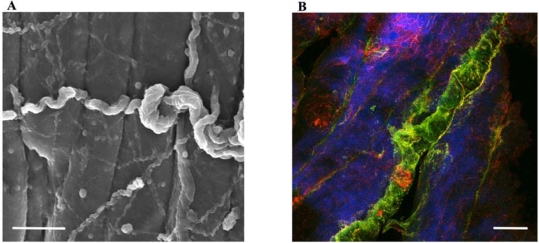
(A) Scanning electron microscope image of a muscle fibre bundle of *M. biceps femoris* of a rabbit, showing the orientation and tortuosity of branching vessels at different hierarchies. Scale bar = 10 μm. (B) Confocal laser scanning microscope image in combination with immuno-labeling for Collagen I and VI illustrating that the arteriole wall is comprised collagen type I (red) and VI (green). On the surface of the neighboring muscle fibres (blue) small reticulin fibres (red) can be distinguished using the collagen I label. Scale bar = 30 μm.

However, though many parameters triggering angiogenesis at the capillary level are well studied, the changes involved in the higher order assembly of the system are lacking. In Figure 2A, a scanning electron microscope image of a muscle fibre bundle in rabbit *M. biceps femoris* shows the orientation and tortuosity of branching vessels at different hierarchies. No obvious orientation of the vessels with respect to muscle fibres can be deduced from this type of image. Indeed, it is not even possible to draw any conclusion about what type of vessel is being looked at, what orientation it is in or where in the hierarchy it is located. Furthermore, no additional fibrillar structures, which could be associated with non-capillary tissue in connective tissue, can be seen. The CLSM image in Figure 2B, which is based on immuno labeling for Collagen I and VI, illustrates the expression of both types in the arteriole wall, but also shows some tethering of the vessel to the neighbouring muscle fibres by fibrils containing Collagen I. If these fibrils facilitate the maintenance of close contact between arterioles and muscle fibers during periods of contraction as well as relaxation, they will confirm a functional advantage in terms of shunt flux and diffusion.

Current knowledge of the structure or architecture of the vascular network is grounded in a number of microscopy techniques, which provide information of a sectional nature, that is to say information gleaned from two-dimensional images or surfaces. The tedious work of combining multiple sections can in principle provide much needed information of the three-dimensional architecture of the vascular network and to this end, some three-dimensional models have been published based on CLSM [31]. However, it is clear that much more work can and must be done in order to clarify the architecture of the network, perhaps requiring the use of novel techniques. A principle issue that still remains is the actual density of arteries, veins, arterioles, venules and capillaries within muscle tissue, put in other words, the exact number of microvessels per fibre cross sectional area calculated in such a way as to minimize multiple counts for the same tortuous vessels.

Developments in the technique of X-ray imaging are currently aimed at improving the resolution at the sub-micron level [49], although this technique has the potential to be of use at much lower resolutions than even this. At least two studies use this technique on the structure of the ancillary network [47]. In the future, the X-ray contrast between capillaries and the surrounding tissue (muscle fibres etc) might be of importance, and perfusion of capillary networks with solutions containing heavy elements might be employed in order to enhance the electron density of the capillary lumen as compared to ordinary tissue containing less dense elements, such as hydrogen, carbon, oxygen, nitrogen and sulphur. Indeed, it seems reasonable to expect that if the resolution limit is pushed further than is currently possible, this technique may provide a valuable new insight into the architecture of the capillary network, quite likely in terms of an actual three-dimensional model. However, this method will only give information of electron densities and does not give any information about the interaction with neighbouring structures or nutrient as well as waste transport. Another promising possibility is the use of ultra-fast high-resolution non-linear microscopy in combination with fluorescence labeling techniques. Functional multi photon microscopy has been proven to be very useful in neurological studies of the brain as well as in developmental studies [42].

### 2.2. The vascular network as an optimization problem (tubes)

Having obtained detailed information of the architecture of the capillary network, one cannot help but question whether it is possible to understand such an architecture in terms of a hypothetical optimization of the functions of the network? In order to address this kind of issue one has first to identify the design parameters that apply and then examine the function of the network. At a very superficial level, the design parameters include the hierarchy of branching, the cross sectional area and length of each level of vessel hierarchy, the angle of branching, the three-dimensional distribution of the finest microvessel within the matrix of muscle and the location of arterioles and venules in space. The blood supply facilitates the delivery of oxygen and nutrients and the removal of waste products to and from the mitochondria of the perfused cells. This supply and removal system should be functional under the conditions prevailing during both a state of rest and non-rest in muscles, as well as being able to accommodate a variable geometry imposed on the system by the contraction of muscle.

Under steady state conditions the total amount of compound *i* (oxygen, nutrient or waste product) that is delivered or removed per unit time, d*n_i_*/d*t*, can be expressed as,

(1) $$\frac{\text{d}n_{i}}{\text{d}t}=\frac{\text{d}V}{\text{d}t}(C_{\text{in}}-C_{\text{out}})$$

where d*V*/d*t* is the blood flow rate through the muscle and (*C*_in_ – *C*_out_) is the concentration difference of component i between the inlet blood and the outlet blood of the muscle. During exercise both the delivery and removal rates may become limiting, affecting the power generated by the muscle. A capillary network that is optimized with respect to a large (*C*_in_ – *C*_out_) across the muscle will favour a greater degree of power generation compared to the blood flow rate. Moreover, an architecture that solely favours a large concentration difference will be based on a number of elements. A high density of the finest capillaries will ensure a good diffusible contact with adjacent muscle fibres and a low flow rate in each of the capillaries, whilst long capillaries will allow greater concentration differences to build up along their length.

**Figure 4.**
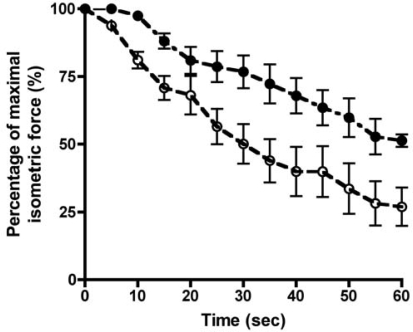
The relation between percentage maximal isometric force *versus* time for stimulated isolated *Soleus* muscles of rats incubated in normal Krebs ringer (for details see Table 1); Controls ( 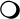; n=8) or AKG treated rats (•; n=4), respectively (see materials & methods for details of the AKG treatment). Isolated muscles were continuously stimulated at 40 Hz for a period of 60 seconds with 32 mA pulses of 1 ms duration, which represents supramaximal, constant-current field stimulation. Force recordings were made *via* a force transducer attached to an A/D converter at a sampling rate of 1000 samples per second. Each point represents mean ± SE. Significant differences between the muscles were found using a Mann-Whitney unpaired two-tailed test (*P*<0.05).

However, an optimization favouring greater concentration differences will result in a state in which not all of the muscle volume contributes equally to force generation. Parts of the muscle volume will be depleted of oxygen or nutrients and will, as a consequence, not contribute to force generation, as is the case for the isolated rat muscle study detailed later in the text and in Figure 4. Clearly an optimization favouring the ratio of power generated to muscle volume and the aforementioned optimization favouring the ratio of power generated to blood supplied are at odds with one another and some compromises have to be made when designing a capillary network.

### 2.3. Cooperative binding of oxygen to haemoglobin – implications for optimal architecture

Interestingly, differences between the mechanisms for transport of oxygen in the blood stream and from the capillaries through the muscle tissue have an impact on both the question of oxygen depleted and less functional regions of muscle. In blood, by far the majority of oxygen is carried by haemoglobin, which binds oxygen through the well-know cooperative binding mechanism (e.g. Hill coefficient, > than one). That is to say that the degree of saturation of oxygen to haemoglobin, *θ*(*p*_O_2__), is a rather steep function of the oxygen pressure and conversely the partial pressure of oxygen *p*O_2_(*θ*), which according to a reversed Hill equation can be expressed as,

(1) $$p{\text{o}}_{2}(\theta )=K{\left(\frac{\theta }{1-\theta }\right)}^{1/n}$$

will only be lowered modestly when haemoglobin releases relatively large amounts of oxygen.

This favours the phenomenon that muscle can utilise relatively large amounts of oxygen at the expense of only a slight drop in the oxygen pressure along the capillaries. The cooperative binding of oxygen therefore helps to diminish the gradient of oxygen pressure along a vessel in which blood is flowing.

For oxygen transport through a tissue, flux can be best described by a modification of Fick’s law,

(1) $$J_{{\text{O}}_{2}}=-D_{{\text{O}}_{2}}\;\nabla p_{{\text{O}}_{2}}$$

where *D*_O_2__is the Krog diffusion coefficient of oxygen. Thus diffusion into mitochondria is expected to be proportional to the *Δp*_O_2, Mitochondria__ between capillaries and mitochondria themselves. The effect of cooperative and non-cooperative binding on the oxygen pressure along the blood flow in the capillaries is illustrated schematically in Figure 2. It is seen that a relatively slight drop in oxygen pressure due to the cooperative binding of oxygen will thus help ensure a good flux of oxygen into the mitochondria, even under situations whereby the blood stream has a relatively low degree of haemoglobin saturation.

Whilst determinants of the diffusional capacity of muscle are complex, structural variables, such as capillary length per fibre volume and capillary-to-fibre surface contact, in combination with functional indices of capillary haemodynamics (e.g. lipid composition affecting membrane resistance), which are considered important determinants of blood-to-myocyte transfer and *vice versa*, lend a critical insight into diffusion capacities of various compounds, nutrients or waste products during exercise. Yet there remains the issue of the relative location of arterioles (nutrient rich) and venules (nutrient depleted) in space. As described earlier, a consequence of such a concentration difference along a capillary length or across a given space is that a driving force for functional diffusion will exist, in this case between arterioles and venules. Furthermore, such a large functional shunt of oxygen, nutrients or waste products would prove far from optimal for the function of a muscle. This functional shunt has been described in the past by a simple linear diffusion mechanism, the argument being that it is roughly proportional to the distance between arterioles and venules. However, here once again, cooperative binding, for example of oxygen to haemoglobin, has the effect of minimizing an oxygen pressure drop along a vessel in which blood is flowing, thereby also lowering any kind of functional shunt between parts of the blood stream with a high degree of haemoglobin saturation and parts of the blood stream with a low degree of saturation (i.e. arterioles *cf.* venules). Moreover, the same can be said of the plasma proteins α_1_ α_2_ and β globulin and albumin with respect to vehicles for the transport of lipids and other substances (e.g. bilirubin, Ca^2+^), respectively, in the blood.

Clearly, the architecture of the capillary network of muscle has to take into account a great many factors, and whilst a number of the functions of the network can be examined hypothetically using simplified equations and laws, the situation is in reality a far more complex one, and deserving of further investigation.

### 2.4. Waste removal in muscles (cables)

The losses in force, velocity and power that define fatigue often lead to serious limitations in muscle and whole body performance [16], making the unequivocal identification of causative agents of this phenomenon an important yet difficult task. Clearly a capillary network, in other words the tubes in our cables, tubes and wires analogy, plays a vital role in muscle contraction and force production, enabling, as it does, a supply of not only oxygen and nutrients needed to sustain the contractions of muscle fibres, but also a waste product/toxin removal route.

Vascular connections that favour the muscle proper (primary circulatory route) and adjacent connective tissue septa (secondary circulatory route) have been reported for several muscles of different species (cat – [39]; monkey – [20]). Importantly, the secondary circulation has been shown to be associated with not only connective tissue but also the interlacing fat deposits one finds in muscle tissue [12]. Thus a change in the primary to secondary circulatory blood flow for muscle favouring the later would prove to be nutritive for connective tissue and the associated adipocytes but relatively less-nutritive for muscle.

Just as extensive circulatory systems have been identified for muscle, it is equally well known that force production is inhibited by a build-up of among others P_i_ and H^+^, two products that are known to change with a period of intense exercise with P_i_ increasing from 5 to 30 or 40 mM, and intracellular pH declining from 7.0 to 6.2 [16, 48]. Moreover, such changes not only affect force production in fast-twitch muscles, they also reduce cross-bridge interactions due to a changed ionic environment [35], leading to a fatigue-induced drop in tension. Furthermore, it has been shown that exposure of muscles to a high extracellular K^+^ concentration gives rise to depolarization of muscle fibres and results in a loss of contractility [16], particularly when this is associated with a simultaneous reduction in extracellular Na^+^ concentration [40]. Elevated magnesium concentrations in plasma also affect muscle function with such symptoms as muscle weakness, fatigue and tremor [51]. Magnesium acts as a calcium-channel blocker and may therefore affect the release of acetylcholine at the neuromuscular junction and in so doing, reduce the endplate potential recorded from a muscle fibre using an intracellular microelectrode [15]. Clearly then there is a great need for an adequate waste removal network if muscles are to maintain prolonged periods of contraction.

### 2.5. Beneficial nutrients and muscles

During exercise, glutamate plays a central role in energy provision, indeed, a consistent finding in several diseases is a reduced skeletal muscle glutamate content [45]. Remarkably, only a few studies have focused on modulation of muscle glutamate status by means of nutritional supplementation where glutamate, glutamine, α-ketoglutarate or branched chain amino acids have been the focus of investigation. Interestingly though, body-builders have used glutamate/glutamine for some time as a dietary additive to improve muscle bulk with some considerable effect. It is also known that during transamination by branched-chain amino transferase (BCAT), glutamate donates an amino moiety to a branched-chain α-keto-acid, forming α-ketoglutarate (AKG). Moreover, enteral feeding of AKG supplements significantly increase circulating plasma levels of such hormones as insulin, growth hormone, and the growth factor IGF-1 [28, 37], endocrine changes which must surely serve to enhance muscle development. However, recent evidence now indicates an effect of AKG directly on collagen [14], and a role as a natural ligand for a G-protein-coupled-receptor (GPR99) specific for AKG, which is expressed in smooth muscle [24]. Besides all of these aspects, one way of decreasing P_i_ is to add alpha-ketoglutarate (AKG) to a muscle, since AKG acts as a phosphate binder [9]. Indeed, over half a century ago, Mudge [38], showed in a study of potassium accumulation by rabbit kidney slices, that oxygen consumption per hour per mg wet weight of tissue increased by 209% *cf*. control levels upon addition of AKG. With every completed TCA cycle, succinyl CoA is converted to succinate and a single P_i_ is converted to a high-energy bond e.g. ATP or GTP. Thus, if addition of TCA intermediates (e.g. AKG) to a tissue causes an increase in metabolism which involves more frequent TCA cycling, one might anticipate a reduction in the level of P_i_ accumulation. It would not seem unreasonable therefore to assume that AKG acting at a number of levels, whether it be in terms of muscle bulk, collagen synthesis and the smooth muscle regulation of blood vessels as well as toxin binding, may be an important natural compound to investigate in association with muscle contraction and force research. Indeed, to this end, a preliminary trial with adult Sprague Dawley rats, administered AKG in the drinking water for a period of 8 weeks, has revealed that the isometric muscle contraction sustainable by isolated slow-twitch soleus muscles from the hind-limb is significantly improved (*P*=0.035; see Figure 4) *cf.* that of control rats. Furthermore, these exciting data lend support to the observation that AKG or succinate have a positive and restorative effect on muscle fatigue in uremic rat skeletal muscle *in vitro* where a benefit is proposed to act at the *in vitro* isolated muscle level by means of phosphate-binding [4].

## 3. Experimental Section

### 3.1. Ethics

The study was approved by the Ethical Review Committee for Animal Experiments at Lund University (Ethical allowance M14-05), and was conducted according to European Community regulations concerning the protection of experimental animals.

### 3.2. Animals

Sprague Dawley rats housed at the animal facilities of the Department of Physiology, Lund University, Sweden were used. The animals were raised under the same conditions with a 12/12 light cycle, and had an average body weight of 345 ± 27g. Rats were fed rodent pellets *ad libitum* (Altromin no.1314 Spezialfutterwerke, Lage, Germany) and given free access to drinking water. The rats were killed by exposure to 95% CO_2_ and cervical dislocation. Rats were killed in accordance with local and national guidelines. A single domestic rabbit carcass, obtained through a commercial source, was dissected *post mortem* and samples of *M. biceps femoris* were removed for subsequent electron microscopy analysis.

### 3.3. Immuno-histochemistry

Muscle samples from the hind legs of adult Sprague Dawley rats were dissected at 24 h *postmortem* and subsequently snap frozen in liquid nitrogen. The samples were then stored at –21 °C until use. The development of type-specific collagen antibodies is dependent on non-denatured three-dimensional epitopes. All the antibodies used in this study had been tested by the producer for cross-reactivity with other collagen types, other serum proteins and non-collagen matrix proteins. The polyclonal antibody, raised against the N terminus of the α1 chain of collagen type I, was purchased from St. Cruz Biotechnology, Inc. (Santa Cruz, CA, U.S.A.). Previously characterized monoclonal antibodies raised against collagen type VI from human fetal membranes and laminin from human placenta were developed by Engvall and obtained from the Developmental Studies Hybridoma Bank under the auspices of the NICHD and maintained by The University of Iowa, (Dept. of Biological Sciences, Iowa City, IA 52242 U.S.A.). The phallotoxin Atto 647N-Phalloidin from Fluka was purchased via Sigma-Aldrich Inc. (St. Louis, MI, U.S.A.). Alexa Fluor 488 donkey anti-mouse IgG, and Alexa Fluor 555 donkey anti-rabbit IgG, were acquired from Invitrogen, Molecular Probes (Eugene, OR, U.S.A.).

### 3.4. Immunofluorescence

Serial sections (20–40 mm thick) were cut using a cryostat (Leica, DE) at −20°C, transferred to Poly-L-lysine coated slides and air-dried. After re-hydration in phosphate-buffered saline (PBS) at pH 7.2, the samples were fixed in 4% paraformaldehyde for 1 h. Sections were blocked overnight in 8% bovine serum albumen (BSA) in PBS buffer (pH 7.2). Incubation with the primary antibodies was performed over a period of 1 h at room temperature. After washing thoroughly with PBS for 1 h, the sections were incubated with secondary antibodies for 1 h at room temperature. The sections were washed again, mounted and stored at 4°C until visualisation. In all cases, control samples were examined, having either been single stained to check for channel cross-talk, or having been incubated with secondary antibodies to determine whether non-specific labeling was present.

### 3.5. Confocal microscopy

A Confocal Laser Scanning Microscope (CLSM) (SP2, Leica Laser Technik GmbH, Heidelberg, Germany) was used. The instrument was equipped with an Argon and a Helium-Neon Laser. In order to avoid false positives by collecting fluorescence emission from adjacent fluorophore channels, a sequential confocal imaging technique was used. Utilizing this technique, the fluorochromes were excited sequentially with individual laser lines and the collected images were then merged during post-imaging processing. The following settings were used: Alexa fluor 488, excitation wave-length 488 nm, emission 498–540 nm; Alexa fluor 555, excitation wave-length 543 nm, emission 543–630 nm; Atto 647 fluor 647 excitation wave-length 633 nm, emission 643–700 nm. During image acquisition each line was scanned 16 times and then averaged. The image shown is the result of a projection from the stack of images in the vertical direction using the maximum intensity mode. The resultant grey-value for each pixel was the highest value present in the column of pixels in the stack with the same (x, y)-position.

### 3.6. Electron microscopy

Samples of rabbit *M. biceps femoris* were fixed in 2.5% cacodylate buffered glutaraldehyde, postfixed in osmium tetroxide (1% aqueous solution), critically point dried and spattered with gold. The specimens were then studied using a FEI scanning electron microscope.

### 3.7. Muscle preparation

Animals in the Control group (n = 5) were given fresh tap water, whilst the treatment group received water enriched with NaOH (1 M) at a rate of 10.5 mL/L to compensate for the acidic effect of the α-Ketoglutarate (AKG) preparation (pH 2.0), a pH correction that did not appear to adversely affect the drinking behaviour of the rats. Animals in the AKG group (n = 5) were administered AKG in the drinking water (2.28 g Na_2_AKG./L - 0.05 g salt per 100 g body wt.) which was provided fresh each day. Treatment was for a period of 8 weeks and no compensation was made to the salt intake of the Controls.

Soleus muscles (weight 97.23± 4.39 mg wet wt.; 84% type I, 7% type IIA, 9% type IID and 0% type IIB fibres [13]) were dissected intact, with both the tendons attached to a portion of the fibula, to facilitate anchoring, and a portion of the Achilles tendon at the opposite end of the muscle. Muscles were securely attached at one end to the force transducer and at the other to two metal pins on the mounting/stimulating block. As described in part [22], isolated rat muscles were mounted vertically in thermostatically controlled chambers, stimulated directly with supramaximal pulses, and force development was recorded. The thermostatically controlled chambers had an internal depth and diameter of 5.5 and 3.2 cm, respectively, holding 44 mL of incubation buffer. The mounting/stimulation block, made of perspex, was 8 cm long, 1.5 cm wide and 1 cm thick. Into this perspex block were inserted two steel pins to hold the isolated muscle and two silver stimulating electrodes (0.88 mm diameter fashioned out of jewelry-grade silver (Dansk Hollandsk Ædelmetal A/S, Copenhagen, Denmark). The stimulator used was a DS3 Isolated Stimulator (Digitimer Ltd.) linked to an 8S MacLab A/D Converter (ADInstruments, UK). As described in part [22], twitches were evoked by supramaximal constant-current field stimulation at 2 Hz, 32 mA, and 2-ms pulse duration. Subsequently, optimally tensioned muscles were stimulated by supramaximal field stimulation at 40 Hz, 32 mA, and 1-ms pulse duration to elicit continuous tetanic contractions in soleus muscles (typically 0.83 ± 0.09 N of isometric force [23]). Twitch and tetanic contractions were measured using a FTO3 force displacement transducer (Grass Instrument, West Warwick, RI) connected to a home-built bridge amplifier and interfaced with a 8S MacLab A/D Converter (ADInstruments, Chalgrove, Oxfordshire, UK).

### 3.8. Incubation solutions

Isometric force was measured under the following conditions. Suspended isolated muscles were placed into the thermostatically controlled chambers at 38°C, that were filled with Normal Krebs Ringer solution and continuously oxygenated with a mixture of 95% O_2_ and 5% CO_2_ (pH 7.4). The chemical composition of the incubation buffer used in connection with isolated soleus muscles of adult rats, comprised in mmol/l: Na^+^ 145, HCO_3_^−^ 25, Ca^2+^ 1.27, Cl^−^ 127, Mg^2+^ 1.2, PO_4_^3−^ 1.2, K^+^ 5.9, Glucose 5, SO_4_^2−^ 1.2 with a mosmol/l value of 313 and a pH of 7.4, and was prepared using the following fine chemicals supplied by Mercks; NaHCO_3_, CaCl_2_, MgSO_4_, KH_2_PO_4_, Na_2_HPO_4_, NaCl, KCl, and D-Glucose, with the latter chemical being added last.

### 3.9. Force measurements

The muscles were suspended vertically in a force-displacement FT03 Grass Force transducer (Grass Instruments, Quincy, MA) setup. The recorded signal was adjusted to zero for muscle slack with the aid of an offset dial mounted on the pre-amplifier unit. Each muscle was exposed to 5 single stimulations where the length of the muscle was adjusted to a length that gave the maximal twitch tension (duration 2 ms, 2 Hz, maximal voltage 90 V, current 32 mA) with the aid of a PowerLab /8S A/D converter (ADInstruments, Chalgrove, Oxfordshire, UK). The transducer scale was set to 200 mV, and a recording speed of 1000 data samples per sec was used. Muscles were left in Normal Krebs Ringer to equilibrate for 30 minutes. Following the equilibration period, the muscle was stimulated continuously to fatigue. Slow-twitch soleus muscles were stimulated at a frequency of 40 Hz. In each experiment, soleus muscles from contralateral legs were measured simultaneously. In order to facilitate comparison of muscles from different rats, the relative decline in force was calculated as a percentage of the maximum isometric force that was reached approx. 1 sec after the onset of stimulation as described previously [22].

### 3.10. Statistics

Data are presented as mean ± SE. Differences between means were tested for statistical significance with the use of a Mann-Whitney unpaired two-tailed test. Differences showing a *P* value >0.05 were considered non-significant.

## 4. Conclusions

There is a clear need for detailed study of the effects of not only metabolites on muscle function, but the precise structural layout and role of the capillary and collagen network that comprises muscles both at rest and during periods of contraction.

In a study of coronary capillaries it was concluded that tethering of these tubes to the surrounding myocytes prevents capillary collapse during systole of the heart [1], and this raises the question as to whether the very fine ”tethers” of collagen shown in Figure 1 perform a similar role in skeletal muscle? The issue of regulation of blood flow through the capillary tubes is another topic much in need of research. Indeed these findings have huge implications for our understanding of nutrient delivery to muscle fibres as opposed to that of connective tissue and adipocytes, information that may well prove critical to aiding our understanding of obesity in man as well as marbling deposition in meat livestock. Elevated plasma K^+^ levels arising with muscle activity are known to affect depolarization of endothelial cells releasing NO etc. thereby inducing relaxation of smooth muscle cells and blood vessels, the underlying events in vasodilation. However, preliminary data suggest that there are cells that respond to elevated K^+^ with a hyperpolarization (Prof. D Klærke, personal communication), apparently also mediated through K^+^-channels, which raises the question as to whether K^+^ sensitivity differences in capillaries may be instigative in the primary and secondary circulatory routes of muscle? Finally, there is the issue of improved muscle performance and force production with AKG supplementation which as yet remains unclear as to whether it is metabolic in nature (e.g. P_i_ scavenging) or structural (e.g. enhanced tethering by collagen ”wires”) in nature.

Interdisciplinary studies addressing this topic are not that common, however, precious data relating to the the role of muscle contraction and force production may even now repose unreported. We urge scholars to think holistically in terms of muscle structure and function and to report detailed microscopy findings alongside research data that focus primarily on the contractile nature of muscle fibres whether it be during normal contractions or in fatigue. In this way we envisage the establishment of a literature database that will be of use in the future study of this important field.

## Figures and Tables

**Figure 3.**
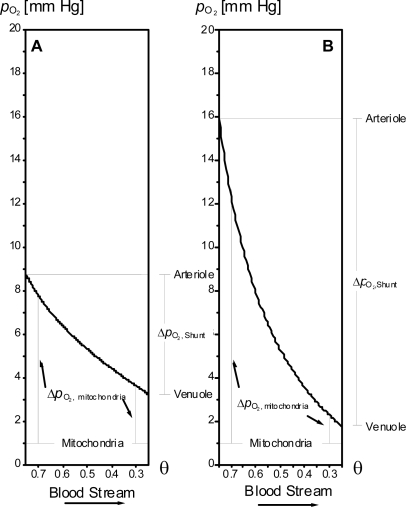
Simplified and schematic illustration of the effect of cooperative and non-cooperative oxygen binding on oxygen pressure along the length of capillaries. The degree of oxygen saturation of haemoglobin, θ, is somewhat arbitrarily taken to be 0.75 and 0.25 for the blood entering and leaving the capillaries. The binding constant is taken to be a realistic value of 5.3 mm Hg. A) Cooperative binding with a realistic Hill coefficient of *n= 2.2*. The variation in oxygen pressure along the length of a stream of blood is relatively little, giving rise to a steady driving force, Δ*p*_O_2, Mitochondria__ along the capillaries and a relatively small driving force for diffusive shunting, Δ*p*_O_2, Shunt__. Cooperative binding constitutes a good foundation for optimization of the capillary architecture. B) A hypothetical situation of non-cooperative binding, *n=*1. The variation of Δ*p*_O_2, Mitochondria__ as well as Δ*p*_O_2, Shunt__ are comparatively large. The optimization of the capillary architecture is a much harder task in this case and in order to avoid regions with low oxygen pressure, as well as in order to minimize the diffusive shunting, the architecture should be changed in order to lower the change of θ . The consequence of this would be that relatively less of the transported oxygen is exploited for contractile force.
